# Developmental stress elicits preference for methamphetamine in the spontaneously hypertensive rat model of attention-deficit/hyperactivity disorder

**DOI:** 10.1186/s12993-016-0102-3

**Published:** 2016-06-17

**Authors:** Jacqueline S. Womersley, Bafokeng Mpeta, Jacqueline J. Dimatelis, Lauriston A. Kellaway, Dan J. Stein, Vivienne A. Russell

**Affiliations:** Department of Human Biology, Faculty of Health Sciences, University of Cape Town, Anzio Road, Observatory, Cape Town, 7925 South Africa; Department of Psychiatry and Mental Health, Faculty of Health Sciences, University of Cape Town, Groote Schuur Hospital, Observatory, Cape Town, 7925 South Africa

**Keywords:** Addiction, Attention-deficit/hyperactivity disorder, Conditioned place preference, Developmental stress, Methamphetamine, Spontaneously hypertensive rat

## Abstract

**Background:**

Developmental stress has been hypothesised to interact with genetic predisposition to increase the risk of developing substance use disorders. Here we have investigated the effects of maternal separation-induced developmental stress using a behavioural proxy of methamphetamine preference in an animal model of attention-deficit/hyperactivity disorder, the spontaneously hypertensive rat, versus Wistar Kyoto and Sprague–Dawley comparator strains.

**Results:**

Analysis of results obtained using a conditioned place preference paradigm revealed a significant strain × stress interaction with maternal separation inducing preference for the methamphetamine-associated compartment in spontaneously hypertensive rats. Maternal separation increased behavioural sensitization to the locomotor-stimulatory effects of methamphetamine in both spontaneously hypertensive and Sprague–Dawley strains but not in Wistar Kyoto rats.

**Conclusions:**

Our findings indicate that developmental stress in a genetic rat model of attention-deficit/hyperactivity disorder may foster a vulnerability to the development of substance use disorders.

## Background

Over recent years a substantial and compelling body of literature has emerged to suggest the importance of gene × environment interactions in the development of psychopathology. Genetic inheritance and environmental factors each account for approximately 50 % of the risk of developing a substance use disorder (SUD) [1]. More specifically, both human and animal studies have identified early life stress and a diagnosis of attention-deficit/hyperactivity disorder (ADHD) as individual risk factors for the development of SUDs [2, 3]. Specific mechanisms involved in such vulnerability have begun to be delineated in humans. A [^11^C]raclopride positron emission tomography study found that a history of childhood adversity increased amphetamine-induced dopamine release in the ventral striatum [4] while data from a functional magnetic resonance imaging (fMRI) study revealed increased limbic area activity after childhood maltreatment in abstinent methamphetamine-dependent individuals [5]. A further fMRI study examining reward-related brain areas found increased activity in the putamen in individuals who had experienced early life adversity whilst activity in the right insula was associated with ADHD symptomology [6]. Specific animal models may be useful in further investigating such mechanisms. Neonatal isolation-induced developmental stress in rats was found to increase cocaine self-administration [7]. In a separate study, developmentally stressed rats displayed a reduced threshold for intracranial self-stimulation of the lateral hypothalamus following amphetamine administration, an indication that the reward-enhancing effect of amphetamine was higher than in controls [8]. Combined these results suggest that early life stress and ADHD may render individuals hyper-sensitive to psychostimulants.

Though the shared pathophysiology underlying developmental stress, ADHD and SUDs is not yet clear, altered dopaminergic transmission affects all three processes and thus has emerged as a likely mechanism. Developmental stress induces long-term changes in the dopaminergic system, reducing dopamine type 2 receptor levels in the nucleus accumbens, which is in turn associated with compulsive drug use and impulsivity [9–11]. Altered striatal concentrations and functional polymorphisms of the dopamine transporter (DAT), the presynaptic transporter responsible for the rapid reuptake of synaptic dopamine and therefore of critical importance in dopaminergic homeostasis, have both been implicated in ADHD [12, 13]. Furthermore, the most widely prescribed medications for the treatment of ADHD are psychostimulants, which exert their effects at dopaminergic synapses [14]. DAT is the molecular target for the psychostimulant drug of abuse, methamphetamine, which binds to DAT and reverses its action essentially causing dopamine to be released into, rather than taken up from, synapses [15]. The subsequent supraphysiological levels of dopamine are responsible for the rewarding properties of drugs and in turn influence a number of dopamine-dependent behavioural processes, which are comorbid with the development of SUDs, such as altered motivation, motor output, impulsivity and reward processing [10, 16].

To further probe the common mechanism underlying developmental stress, ADHD and SUDs, we previously used in vivo chronoamperometry to examine the effect of developmental stress, via maternal separation (MS), on striatal dopamine clearance in an animal model of ADHD. The spontaneously hypertensive rat (SHR), in comparison to its normotensive progenitor strain the Wistar Kyoto rat (WKY), is a well-validated animal model of ADHD [17–19]. Our preliminary results indicated that MS delayed the clearance of ejected dopamine in SHR suggesting reduced DAT efficiency [20]. A further study examining the effect of cocaine, a psychostimulant and potent DAT inhibitor, in this model found that the cocaine-induced delay in dopamine uptake was exacerbated by MS in SHR resulting in a prolonged elevated dopamine concentration [21]. Given these results, we hypothesise that the observed decrease in DAT efficiency in MS SHR will translate into an increased preference for the psychostimulant methamphetamine, a drug with a high potential for dependence and abuse [22]. We examined this proposal using SHR, WKY and an additional comparator strain, Sprague Dawley (SD), to control for the putative depressive/anxious phenotype of WKY, which might influence our results [23, 24]. This study used adolescent rats, an age associated with the onset of drug use and prior to the full development of elevated blood pressure in SHR [25, 26]. We employed the conditioned place preference (CPP) test, a classical conditioning paradigm that pairs drug exposure with one of two visually and tactilely distinct compartments to determine whether drug administration can overcome an innate initial preference. Successful pairing of a drug with the non-preferred compartment and a change in location preference is used as a proxy for the rewarding properties of the drug [27].

## Methods

### Animals

SHR (Charles River Laboratories, Wilmington, MA, USA), WKY (Harlan Laboratories, Bicester, UK) and SD (Charles River Laboratories, Wilmington, MA, USA) rats were obtained from strains maintained at the University of Cape Town Research Animal Facility. The decision to source WKY from Harlan, UK, rather than Charles River Laboratories, was based on research suggesting that they are the most appropriate behavioural and genetic control [24]. Rats had ad libitum access to water and standard rat chow and were housed in clear Perspex cages with wood chip bedding in a facility maintained at 21–23 °C with a 12/12 h light/dark cycle (lights on at 06h00). All experiments were authorised by the University of Cape Town Faculty of Health Sciences Animal Ethics Committee under application 011/047 and conformed to local and international standards set out for the care and use of animals for scientific purposes [28, 29].

### Maternal separation

The MS paradigm was performed as previously described [30]. Briefly, male and female rats were pair bred in the University of Cape Town Human Biology satellite animal facility and the day of birth of the resulting litters was designated as postnatal day 0 (P0). On P2, the dam was removed from the cage and the number and sex of the pups was determined. In order to maintain uniformity of care, litters were culled to 8 pups with males preferentially selected for. However, a minimum of 2 female pups were retained in each litter to control for possible altered maternal behaviour and subsequent anxiety in offspring due to varying litter gender composition [31, 32]. Dams of non-separated (nMS) litters were subsequently returned to the home cage and remained with the litter in the animal facility until weaning. Conversely, on P2 MS litters were removed from the dam to a separate room maintained at 31–33 °C with infrared heating lamps. Three hours later the litters were returned to the animal facility and the dam returned to the home cage. This separation paradigm occurred between 09h00 and 13h00 over 13 days from P2 to P14. Cleaning of cages and the initial handling of pups on P2 was consistent across MS and nMS groups to ensure that potential differences would be due to the effect of the separation paradigm. On P21 litters were weaned and male rats we co-housed (2–4 rats/cage) for the remainder of the project. No more than 2 rats from any one litter were assigned to an experimental group so as to avoid potential confounding litter effects.

### Conditioned place preference

The CPP paradigm was performed over the course of 7 days (P54–P60) in male adolescent rats, thereby corresponding with the most common age of onset for SUDs in humans [33]. This compressed protocol consisted of 3 preconditioning, 3 conditioning and 1 probe trial day. Briefly, a square black Perspex box (43 cm length × 50 cm height) was equally divided by a central partition to produce one chamber with a grid floor and thin vertical white stripes on the walls and a second chamber with unadorned walls and a smooth floor. Rats were allowed to freely explore the apparatus for 30 min during preconditioning, which was performed over the course of 3 days to compensate for the increased exploratory drive and preference for novelty in SHR as well as potential anxiety in WKY [34]. The compartment in which rats spent the most time on the 3rd day of testing was designated as the preferred compartment. The conditioning period was composed of 2 × 1-h trials per day with a vehicle (0.9 % saline administered via intraperitoneal injection at 1 ml/kg volume) injection paired with the preferred compartment, and a methamphetamine (Sigma-Aldrich, St Louis, MO, USA) injection (1.5 mg/kg in 0.9 % saline administered via intraperitoneal injection at 1 ml/kg) paired with the non-preferred compartment. These 2 trials were separated by at least 3 h to allow sufficient time for memory formation with the non-drug pairing conducted first to prevent the association of potential withdrawal effects with the subsequent trial [27]. The selected dose of methamphetamine (1.5 mg/kg calculated as a free base) was based on 3 factors: successful CPP in SHR following conditioning with a 1.25 mg/kg dose; the failure to find an effect of MS on place preference in SD rats administered a 1.0 mg/kg dose; and the need to avoid potential neurotoxic side effects associated with a higher dose, which might reduce locomotor activity due to depressive effects [35–38]. As caudate putamen methamphetamine concentrations peak between 30 and 60 min post intraperitoneal injection, rats were injected 10 min prior to the onset of conditioning trials to ensure that peak cerebral concentrations of methamphetamine were reached within the 1 h conditioning trial [39]. On the final day of testing, P60, rats were exposed to a 30 min probe trial during which they were allowed to freely explore the apparatus. Behaviour was recorded using a Soni Handicam DCR-SX 83E and time spent in each compartment as well as locomotor activity were analysed using Noldus Ethovision XT 7.0 (Noldus Information Technology, Wageningen, Netherlands). This experimental design produced 6 final groups: nMS SHR (n = 13), MS SHR (n = 11), nMS WKY (n = 10), MS WKY (n = 13), nMS SD (n = 13) and MS SD (n = 10).

### Statistical analyses

All data were tested for normal distribution using a Shapiro–Wilk W test. Baseline activity data over the course of the 3 preconditioning days was analysed to check for strain and stress effects. The time spent highly mobile (defined as the period of time during which the area detected as the animal changes by at least 60 % per second) was non-parametrically distributed and therefore tested for potential strain × stress effects using a Kruskal–Wallis test with multiple comparisons of mean ranks with Bonferroni adjustment as a post hoc test. To check for differences in the initial strength of compartment preference, the duration spent in the non-preferred compartment on the third day of preconditioning was subjected to a factorial ANOVA with strain and stress as categorical predictors. Significant differences were further investigated using a Tukey post hoc test. Methamphetamine preference scores were calculated by subtracting the time spent in the non-preferred compartment on the third day of preconditioning from the time spent in the same compartment during the probe trial. Therefore a positive value, i.e. increased time spent in the non-preferred compartment following methamphetamine conditioning, was taken as an indication of increased preference for the drug-paired compartment. Preference scores were normally distributed and thus analysed using a factorial ANOVA with strain and stress as categorical factors. Significant differences between groups were probed using a Tukey post hoc test. To determine which groups displayed behavioural sensitisation to methamphetamine, the total distance covered and the time spent highly mobile on the first and third days of conditioning were compared. As these data were non-parametrically distributed, they were analysed with a Wilcoxon Matched Pairs Test. To check for strain × stress effects on sensitisation, we subjected the mobility data to an aligned rank transform for nonparametric factorial analyses [40]. This preprocessing allows common ANOVA procedures to be used to investigate interaction effects in repeated measures non-parametrically distributed data. All statistical analyses were performed using Statistica 13 (Statsoft, Dell Software, Tulsa, OK, USA) and an α value of 0.05 was used to determine significance. Graphs were generated using GraphPad Prism 6.0 (GraphPad, La Jolla, CA, USA).

## Results

**Fig. 1:**
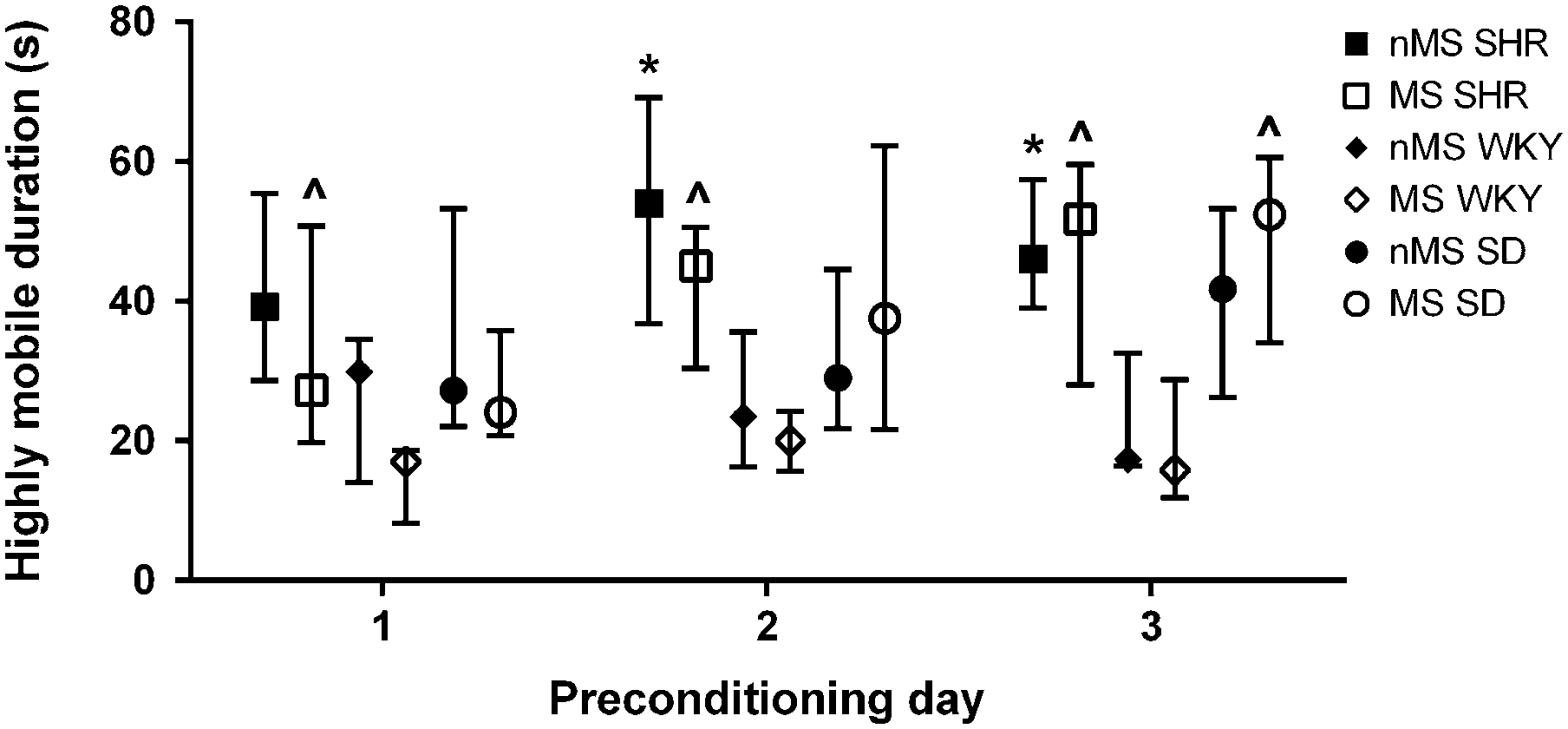
SHR and WKY rats exhibit baseline differences in the time spent highly mobile during preconditioning. ^MS WKY spent less time highly mobile compared to MS SHR on days 1 through 3 of preconditioning and compared to MS SD on the third day of preconditioning (p < 0.05, Bonferroni post hoc test). *nMS WKY spent less time highly mobile than nMS SHR during the second and third days of preconditioning (p < 0.05, 613 Bonferroni post hoc test). Data are displayed as median and interquartile range

The time spent highly mobile was recorded over the 3 preconditioning days and analysed to check for differences in baseline locomotor activity. Kruskal–Wallis tests revealed significant strain × stress effects on the time spent highly mobile on each of the 3 days [day 1 H(5,N = 70) = 23.47, p < 0.001; day 2 H(5,N = 70) = 22.43, p < 0.001; and day 3 H(5,N = 70) = 26.27, p < 0.001] (Fig. 1). Post hoc analysis revealed that MS WKY spent less time highly mobile than MS SHR across all 3 days (day 1 p = 0.022, day 2 p = 0.020, and day 3 p = 0.013). MS WKY also spent less time highly mobile than MS SD on the third day of preconditioning (p = 0.009). Further differences between SHR and WKY rats were also found on days 2 and 3 of preconditioning where nMS SHR spent more time highly mobile than nMS WKY (p = 0.020 and p = 0.039 respectively).

**Fig. 2:**
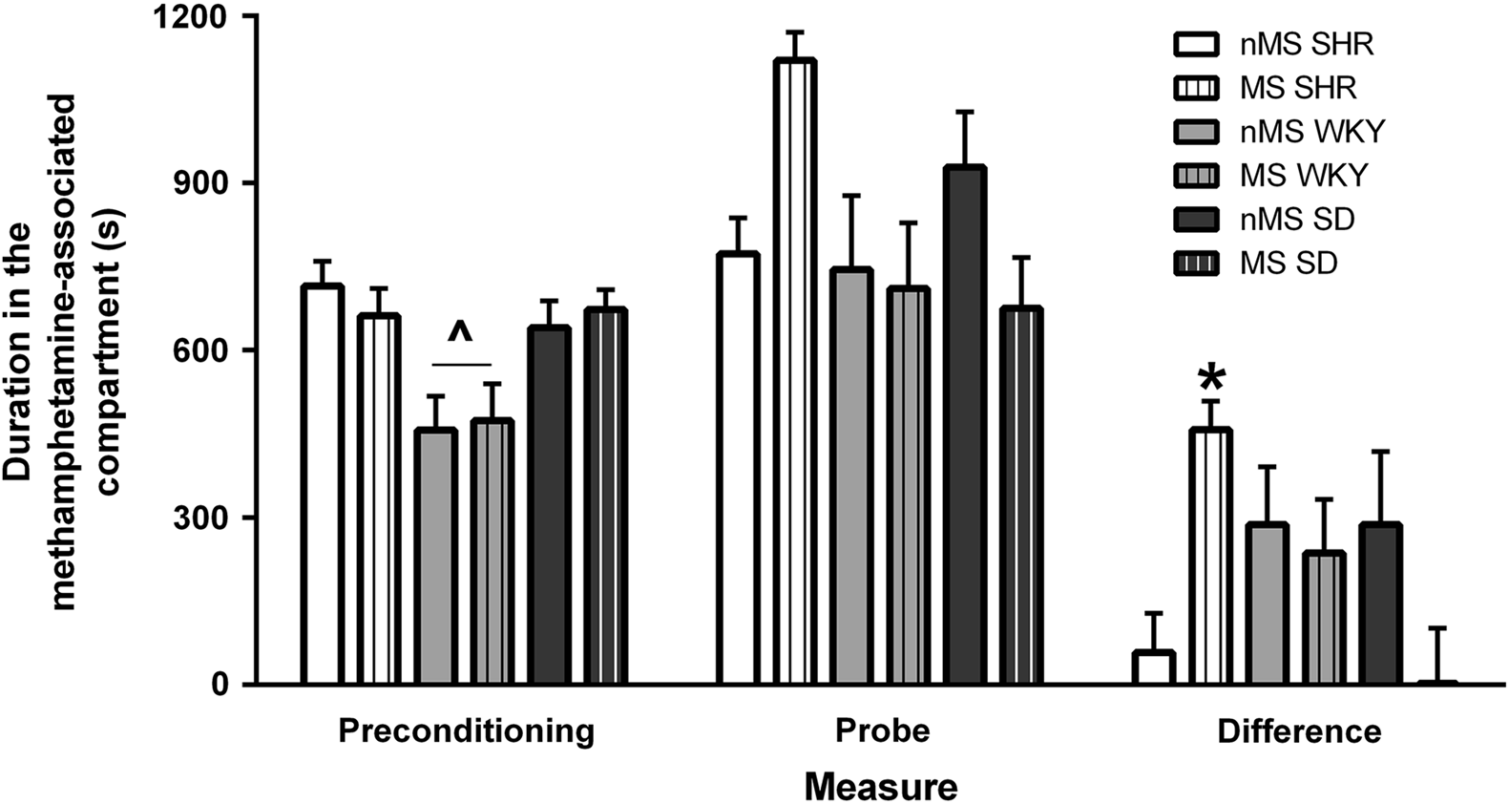
MS SHR displayed preference for the methamphetamine-paired compartment. *The difference in time spent in the non-preferred/methamphetamine-paired compartment between the third day of preconditioning and the probe trial was greater in MS SHR than nMS SHR and MS SD (p < 0.05, Tukey post hoc test). ^WKY displayed a stronger initial preference for the preferred compartment than both SHR and SD (p < 0.05, Tukey post hoc test). Data are displayed as mean ± SEM

The change in time spent in the non-preferred compartment following methamphetamine administration was analysed by factorial ANOVA and revealed a significant strain x stress interaction (F_(2,64)_ = 6.45, p = 0.003). A post hoc test indicated that MS SHR spent a longer period in the methamphetamine-paired compartment compared to both nMS SHR and MS SD (p = 0.049 and p = 0.029 respectively) (Fig. 2). MS did not alter preference for the methamphetamine-paired compartment in either WKY or SD and no strain difference was found within the nMS group. The strength of initial compartment preference was analysed by factorial ANOVA and revealed a significant strain effect (F_(2,64)_ = 10.36, p < 0.001). A Tukey post hoc test indicated that WKY spent more time in the preferred compartment initially than both SHR and SD (p < 0.001 and p = 0.002 respectively) (Fig. 2).

**Fig. 3:**
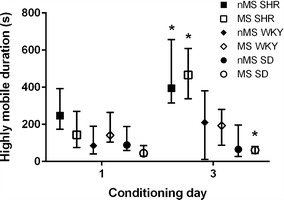
SHR spent more time highly mobile after repeated methamphetamine administration. *nMS SHR, MS SHR and MS SD spent more time highly mobile on the third day of conditioning than on the first day (p < 0.05, Wilcoxon matched pairs test). Results are displayed as median and interquartile range

**Fig. 4:**
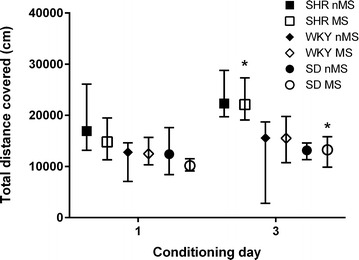
MS increased locomotor activity after repeated methamphetamine administration in SHR and SD rats. *MS SHR and SD travelled further on day 3 than on day 1 (p < 0.05, Wilcoxon matched pairs test). Results are displayed as median and interquartile range

Potential sensitisation to methamphetamine was determined by comparing locomotor activity data obtained on the first and third days of methamphetamine conditioning. A Wilcoxon matched pairs test indicated an increase in time spent highly mobile on the third day of conditioning for all groups (T_(n=70)_ = 619.5, p < 0.001). This difference was significant in nMS SHR (T_(n=13)_ = 8.0, p = 0.009), MS SHR (T_(n=11)_ = 2.0, p = 0.006) and MS SD (T_(n=10)_ = 4.0, p = 0.028) (Fig. 3). A further Wilcoxon matched pairs test revealed a significant effect of methamphetamine on total distance covered in all groups (T_(n=70)_ = 640.0, p < 0.001), which was due to MS SHR and MS SD covering significantly greater distances on the third day of conditioning (T_(n=11)_ = 8.0, p = 0.026, and T_(n=10)_ = 6.0, p = 0.028 respectively) (Fig. 4). Repeated measures ANOVAs of aligned rank transformed data revealed no significant strain × stress effects for either the duration highly mobile or the total distance covered.

## Discussion

In this study we sought to investigate whether our earlier observation of delayed dopamine reuptake in MS SHR translates into increased preference for the psychostimulant methamphetamine measured using a CPP paradigm. Consistent with our hypothesis, MS SHR displayed increased preference for the methamphetamine-paired compartment compared to both nMS SHR and MS SD. The failure of MS in SD rats to increase methamphetamine preference is consistent with a previous study, in which nMS and MS male SD rats were exposed to 2 conditioning stages (P33–36 and P39–42) with methamphetamine (administered at 1 mg/kg) yet did not differ in their preference for methamphetamine at P37, P43 or P50 [35]. The finding that WKY did not develop methamphetamine CPP is also in keeping with a previous study that showed that rats with high anxiety exhibit less 50 kHz ultrasonic vocalizations—a marker of positive affect [41]. We also investigated whether there were any differences in the initial magnitude of compartment preference and determined that WKY showed a more robust preference for the saline-paired compartment during preconditioning than both SHR and SD rats. Perhaps more importantly for the current results, there was no difference in the strength of compartment preference between nMS and MS SHR. Combined these results suggest that the influence of developmental stress on drug preference is dependent on genetic predisposition.

The failure of nMS SHR to show a robust preference for the methamphetamine-associated compartment is perhaps surprising given the association between ADHD and drug abuse [42]. However, we maintain that previous studies which found that SHR exhibit a preference for psychostimulants differ from our own in several important methodological aspects including the source of the SHR used and the age at testing. For example, several studies in SHR have found positive preference for methamphetamine (1.25 and 5.0 mg/kg) as well as the psychostimulants amphetamine (5 mg/kg) and methylphenidate (1.25, 5.0 and 20 mg/kg) [37, 38, 43, 44]. Importantly, these studies made use of SHR obtained from Charles River Japan as opposed to the current study which sourced SHR from Charles River USA, a noteworthy distinction given evidence that suggests that SHR from different vendors may display different behavioural characteristics [24]. Furthermore, in the aforementioned studies, the age of the rats at the time of behavioural testing differed by at least a week compared to our protocol. Though a small difference, age has previously been suggested to influence the response to psychostimulants in SHR [45, 46]. A further possible reason for the failure to find methamphetamine CPP in nMS SHR in the current study lies in the behaviour of the strain. Previous research has indicated that, congruent with the hyperactive phenotype, SHR continue to display increased locomotor activity in familiar environments [47]. Indeed, an analysis of preconditioning baseline activity revealed that SHR were more active than their WKY counterparts. It is therefore possible that the rewarding effects of methamphetamine were insufficient to prevent nMS SHR from exploring both chambers of the apparatus during the probe trial.

A repeated measures analysis of the locomotor activity on the first and third days of conditioning found no significant strain × stress effects on the magnitude of sensitisation. However, a matched pairs comparison of locomotor activity on the first and third days of conditioning indicated that nMS SHR, MS SHR and MS SD spent more time highly mobile after repeated methamphetamine administration, which translated into an increased total distance covered in the latter two experimental groups. As these results measure the change in locomotor activity due to methamphetamine, they are not influenced by the higher baseline activity levels of SHR. This suggests that SHR, and in particular MS SHR, display an increased sensitisation to methamphetamine i.e. repeated exposure produces an increased stimulant drug response that is associated with increased motivation to consume the drug [48]. The current study made use of ambulatory activity to measure locomotor sensitisation. However, repeated exposure to psychostimulants may also lead to the development of repetitive motor behaviours or stereotypies such as repeated sniffing, rearing, and head and mouth movements [49]. Previous research using Lewis and Fischer 344 rats has indicated that the developmental time course of stereotyped behaviour in response to methamphetamine may differ between strains [50]. Furthermore, a study comparing the behavioural responses of 6 week old SHR and WKY to d-amphetamine found strain differences in the types of stereotypic movement produced [51]. It is therefore possible that an analysis of stereotyped behaviour in the current study may have revealed further strain and/or stress effects, including sensitisation to methamphetamine in WKY.

Comparison of the methamphetamine preference and sensitisation results reveal a conflict insofar as methamphetamine elicited preference in MS SHR whilst increasing sensitisation in nMS SHR, MS SHR and MS SD. This apparent contradiction between psychomotor and reward responses to psychostimulants has also been found in previous studies. One such investigation examined the effect of self-administration duration on drug-primed reinstatement and behavioural sensitisation [52]. Rats that advanced from short to extended access durations (1 vs. 6 h) escalated their cocaine consumption during the first hour of their trials such that they infused more cocaine in that period than their short access (1 h only) counterparts [52]. However, this group that displayed increased drug self-administration, a measure of drug preference, did not differ in locomotor sensitisation to the drug. Further, in a study that assessed locomotor activation, sensitisation and place preference in response to cocaine in 6 mouse strains, the authors failed to find locomotor sensitisation in certain strains that displayed drug CPP [53]. This led the authors to hypothesise that the psychomotor and rewarding effects of drugs may be served by distinct mechanisms. Extending this hypothesis to our own results, it is possible that developmental stress may exert different effects on these mechanisms within the strains we tested. Support for this explanation is provided by previous research suggesting that the time course of dopaminergic development differs between SHR and comparator strains. In an in vitro autoradiography study assessing striatal dopaminergic development in pre- and post-hypertensive (2 and 15 week old respectively) SHR and WKY, DAT was elevated in the caudate putamen of SHR at both developmental stages [54]. In addition, SHR putamen followed a lateral-to-medial DAT gradient during early development and displayed elevated dopamine type 1 receptor concentrations compared to WKY by 15 weeks [54]. A further study examining [^3^H]dopamine uptake into synaptosomes prepared from WKY and SHR (at 1, 2, 3, 6, 8 and 10 weeks of age) found that the rate of dopamine uptake in the prefrontal cortex was persistently lower in SHR from 2 weeks onwards and transiently lower in the striatum at the 6 week time point [55]. Though we were unable to find reports evaluating dopaminergic development in SHR and SD strains, the comparisons between SHR and WKY suggest possible strain differences in the developmental course of multiple dopaminergic pathways. It is therefore possible that the effects of MS may be region specific in different strains, based on the developmental stage of a particular brain area. In this way, MS may affect brain areas responsible for both locomotor activity and reward in SHR, whilst having a more anatomically restricted effect in influencing only locomotor activity in SD.

The potential of early life stress to exert long term changes on neurophysiology is well-recognised [56]. Of relevance to our MS model, immunohistochemical studies have indicated that development of the striatal dopaminergic system continues postnatally with an increase in axospinous connections and a decrease in axodendritic and axosomatic synapses [57]. The overall reduction in total dopaminergic synaptic density bears a closer resemblance to the adult profile [57]. Disruption during this critical window may produce psychopathology associated with dopaminergic dysfunction including ADHD and SUDs [58–60].

Both increased locomotor activity in response to methamphetamine and successful place preference are dependent on dopamine and as such these two measures can be used as a proxy of increased extracellular dopamine concentration [16, 61]. Given the preference for and sensitisation to methamphetamine displayed by MS SHR, it is likely that developmental stress in SHR increased the extracellular dopamine concentration in the brain areas serving these functions. This would also be in keeping with our previous in vivo chronoamperometric studies where the DAT-mediated clearance of dopamine was delayed by MS in SHR [20, 21]. We hypothesise that the dose of methamphetamine used in the study (1.5 mg/kg) was non-saturating i.e. the number of DATs unimpeded by methamphetamine was reduced in MS SHR compared to controls. This scenario could result in a similar dopamine release between groups but prolonged elevated synaptic dopamine in MS SHR due to reduced DAT-mediated clearance. This is consistent with evidence for altered DAT function and responsiveness to psychostimulants in ADHD as found by Stein et al., where children diagnosed with ADHD and possessing the 9/9 repeat DAT allele were less sensitive to the effects of the therapeutic psychostimulants on measures of hyperactivity and impulsivity [62]. However, further experiments measuring methamphetamine-induced dopamine release and DAT-mediated reuptake would be required to either support or refute this proposed mechanism. This hypothesis would also be further refined by an experiment that examines whether MS affects DAT expression differently in SHR compared to WKY and SD.

## Conclusions

Our finding of preference for the methamphetamine-associated compartment in MS SHR strongly supports our previous chronoamperometric findings of reduced DAT-mediated dopamine clearance in MS SHR. Given the high cost of SUDs to individuals and society, such mechanistic insights are important in understanding how a diagnosis of ADHD and a history of developmental stress may increase the risk of developing SUDs. Furthermore, these results again reinforce the importance of gene × environment interactions in influencing psychopathology.

## Abbreviations

ADHD: attention-deficit/hyperactivity disorder
CPP: conditioned place preference
DAT: dopamine transporter
fMRI: functional magnetic resonance imaging
MS: maternally separated
nMS: non-maternally separated
P: postnatal day
SD: Sprague–Dawley
SHR: spontaneously hypertensive rat
SUD: substance use disorder
WKY: Wistar Kyoto
